# Electron cryomicroscopy as a powerful tool in biomedical research

**DOI:** 10.1007/s00109-018-1640-y

**Published:** 2018-05-05

**Authors:** Dennis Quentin, Stefan Raunser

**Affiliations:** Department of Structural Biochemistry, Max Planck Institute of Molecular Physiology, Otto-Hahn-Str. 11, 44227 Dortmund, Germany

**Keywords:** Electron cryomicroscopy, Cryo-EM, Biological macromolecule, Biomedical research, Drug design

## Abstract

A human cell is a precisely regulated system that relies on the complex interaction of molecules. Structural insights into the cellular machinery at the atomic level allow us to understand the underlying regulatory mechanism and provide us with a roadmap for the development of novel drugs to fight diseases. Facilitated by recent technological breakthroughs, the Nobel prize-winning technique electron cryomicroscopy (cryo-EM) has become a versatile and extremely powerful tool to solve routinely near-atomic resolution three-dimensional protein structures. Consequently, it has become the focus of attention for structure-based drug design. In this review, we describe the basics of cryo-EM and highlight its growing role in biomedical research. Furthermore, we discuss latest developments as well as future perspectives.

## Basics of single-particle cryo-EM

The function of cellular macromolecules is closely related to their three-dimensional architecture. Detailed structural insights help us not only to understand the mechanism underlying cellular processes, but provide us with a topological map for the development of potential therapeutic compounds.

Over the last 50 years, the determination of atomic protein structures has been dominated primarily by X-ray crystallography and, to some extent, by NMR spectroscopy. Although both methods have contributed enormously to our current molecular understanding of biological processes, they also come with drawbacks. For X-ray crystallography, biomolecules have to be crystallized. Obtaining well-diffracting crystals, however, is often challenging, time-consuming, and, in several cases, impossible. Although NMR does not require crystals, it is only practicable for molecules with low molecular weight, usually below 50 kDa. Single-particle cryo-EM has been an alternative for solving the structure of large protein complexes and filaments for decades. It does not depend on crystals, and protein structures can be determined rather quickly. However, until recently cryo-EM structures were limited in resolution and did not allow building atomic models. Due to important technological advances, single-particle cryo-EM has caught up rapidly in recent years [1]. Now, high-resolution structures can be obtained routinely, rendering cryo-EM to a major technique in structural biology.

In transmission electron microscopy (TEM), accelerated electrons pass through and interact with the specimen. The interference between scattered and non-scattered electrons results in the so-called phase contrast and image formation. Because electron microscopes require high vacuum, living cells or more generally hydrated samples cannot be examined by this method at room temperature.

In cryo-EM, this problem is solved by embedding the samples in amorphous ice through plunge-freezing in liquid ethane, a sample preparation method developed by Dubochet and colleagues in the early 1980s [2]. When imaged at cryogenic temperatures (77 K), the vapor pressure of the so-called vitrified sample is low and the proteins can therefore be imaged in their hydrated state.

**Fig. 1:**
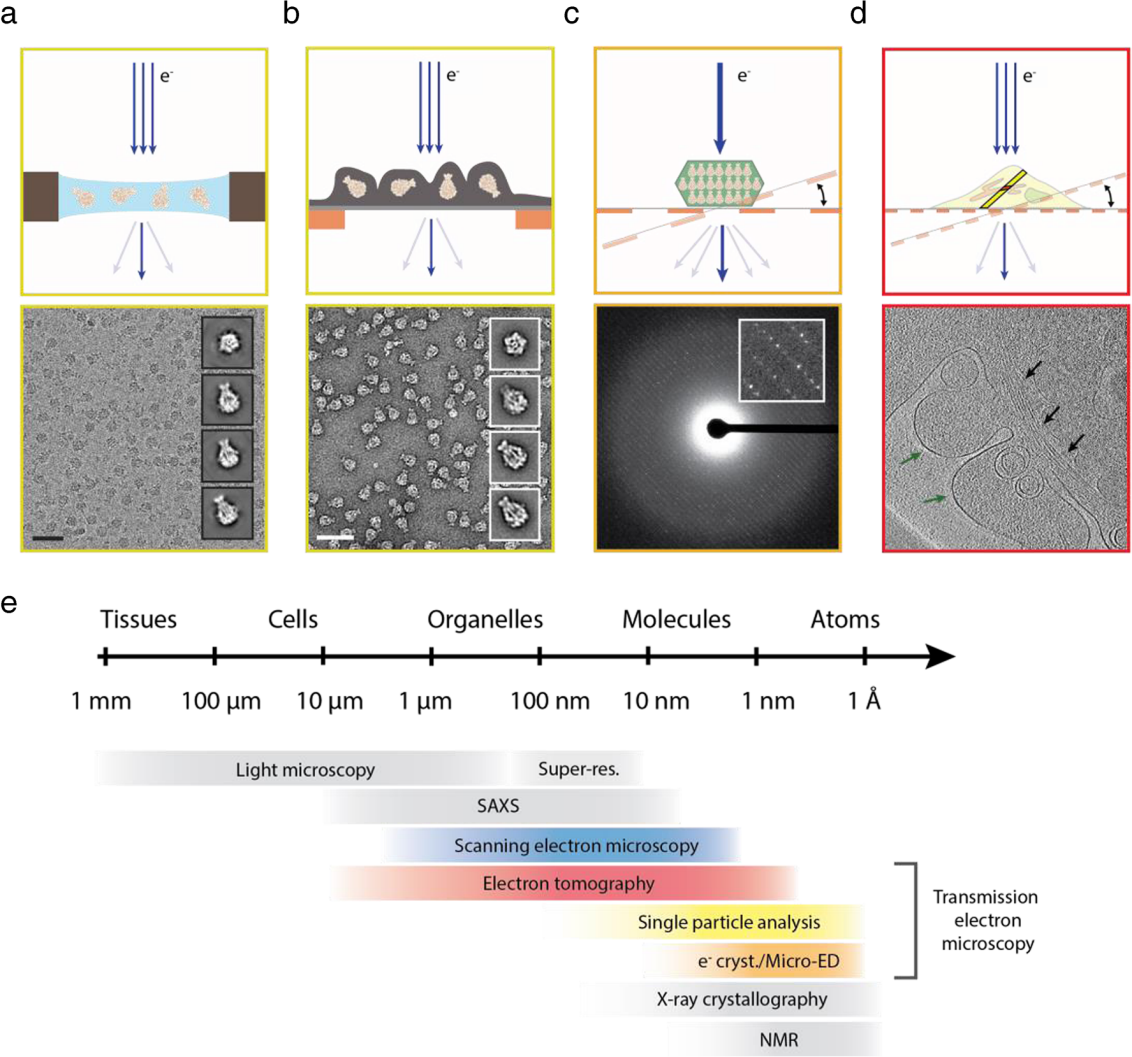
The most common cryo-EM techniques. Schematic drawing in (**a**) to (**d**) illustrates principles of the most popular cryo-EM methods in the upper panel and shows corresponding raw data in the lower panel. (**a**) Single-particle cryo-EM: particles are embedded in a thin layer of amorphous ice. Resulting representative class averages are shown as insets on the right. Scale bar, 50 nm. (**b**) Single-particle negative-stain EM: particles are embedded in a layer of heavy metal salts to increase the weak contrast of biological materials. Resulting representative class averages are shown as insets on the right. Scale bar, 50 nm. (**c**) Micro-ED: small 3-D crystals are hit with a focused electron beam and diffraction patterns are recorded at different tilt angles. Inset shows a small section of the diffraction image with individual diffraction spots at higher magnification. The electron diffraction image was kindly provided by T. Gonen, Janelia Research Campus. (**d**) Cryo-ET: the specimen is tilted within the microscope and images at different angles are recorded. A tomographic slice shows the cellular periphery with microtubule bundles (black arrows) and plasma membrane (green arrows). (**e**) Resolution range coverage of various methods in structural biology. Color code used for the TEM-based methods corresponds to (**a**)–(**d**). Yellow: single-particle analysis; orange: electron crystallography/micro-ED; red: electron tomography

The contrast increases with increasing atomic number. Therefore, TEM images of biological matter, which is composed mainly of light elements, such as carbon, oxygen, and hydrogen, have relatively low contrast. Biological samples are also very vulnerable to electron-beam damage [3]. Therefore, the cumulative electron dose has to be kept low during image acquisition in order to reach atomic resolution, even further decreasing the single-to-noise ratio of the images. Consequently, the images of many proteins have to be recorded and overlaid to increase the signal-to-noise ratio. In addition, since cryo-EM images represent two-dimensional (2-D) projections of a three-dimensional (3-D) object, many images of a protein in different orientations are needed to computationally reconstruct its 3-D structure [4] (Fig. 1a). Joachim Frank has been the driving force behind the development of computer-based image processing over the years.

Because of the low contrast of biological specimens in cryo-EM, the thickness of the surrounding amorphous ice should be as thin as possible. At the same time, there should be as many particles as possible in an image in order to increase the size of the dataset and decrease the amount of measuring time. The sample preparation technique is therefore crucial for the success of the analysis and normally comprises the following workflow. Initially, the quality and suitability of the sample is checked using negative-stain EM [5]. For this, the protein is embedded into a layer of heavy metal salts, mostly uranyl formate (Fig. 1b). This increases the contrast and is easy to use. Although the sample is dehydrated and flattened during sample preparation, negative-stain EM is ideal for the initial examination of protein samples.

Suitable samples - i.e., homogeneous, pure, and stable protein complexes - are then vitrified for cryo-EM. The samples are applied to a carbon-coated copper, molybdenum, or gold grid. The carbon film contains holes of regular size, over which the sample is spread. Before plunging into liquid ethane, the excess protein solution is blotted away using filter paper. The parameters that have to be optimized during vitrification are the blotting time; humidity; type of filter paper; protein concentration; different grid types (hole size, metal support, support layer); the addition of additives, such as detergents to facilitate spreading of the sample; multiple blotting rounds to saturate the surrounding carbon with protein; and the buffer composition [6–8]. Some proteins cannot be obtained at sufficiently high concentration to directly embed them into amorphous ice. In this case, an additional support layer of carbon or graphene oxide can be used that spreads over the holes and to which the proteins adhere [9]. This decreases the necessary protein concentration by up to two orders of magnitude. The optimization of freezing conditions is often laborious but key to successful structure determination at high resolution by cryo-EM.

Images are taken under cryogenic conditions using a state-of-the-art high-end transmission electron microscope equipped with a field emission gun and direct electron detectors (DED). Single particles are digitally extracted from TEM images and processed resulting in 3-D reconstructions at up to 1.8-Å resolution [10]. Reconstructions at near-atomic resolution (a term used to describe cryo-EM densities with resolutions between ~ 2.5 Å to ~ 4 Å) allow *de novo* building of atomic models and their biochemical interpretation.

## New developments paved the way for near-atomic resolution

A number of developments over the years enabled cryo-EM to enter the current era of near-atomic structure determination. The performance of transmission electron microscopes has steadily improved, resulting in, for example, high-performance field emission guns, stable stages, and automated data acquisition over extended periods of time. Especially the development of direct electron detectors (DED) has been a game-changer. In contrast to previous detection technologies, namely photographic film and charge-coupled device (CCD) cameras, in DEDs, primary electrons are directly converted into electrical signals, increasing their detection quantum efficiency (DQE) in all frequencies and consequently the signal-to-noise ratio of recorded images [11]. In addition, DEDs have a high read-out speed, allowing the recording of movies instead of single images. Since the signal-to-noise ratio of the single movie frames is relatively high, they can be aligned to correct the movement of the particles in the amorphous ice during recording [12]. The result is a tremendously increased resolution.

**Fig. 2:**
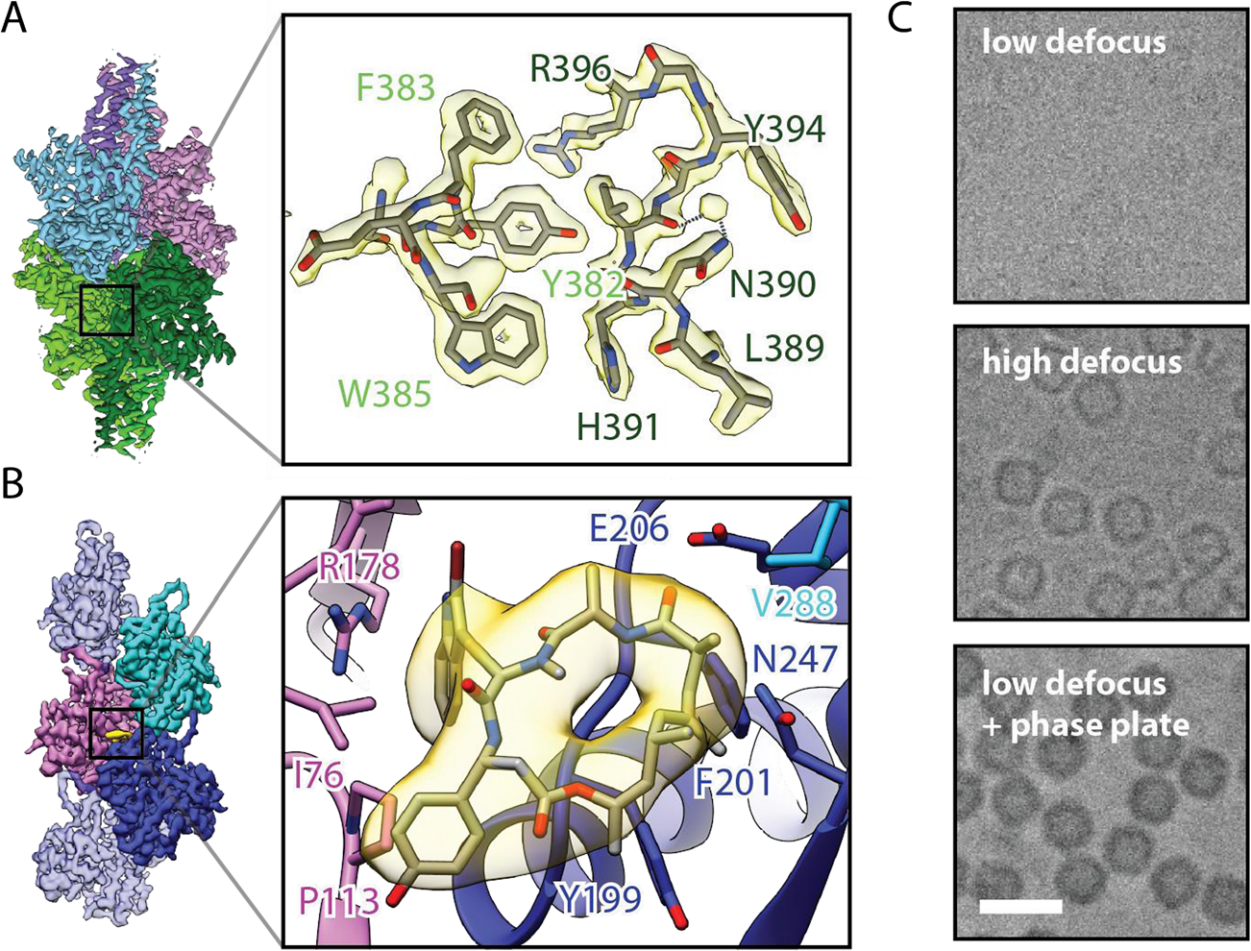
High-resolution cryo-EM as tool for structure-based drug design. (A) Electron density map of the 1.8-Å structure of glutamate dehydrogenase, showing that single-particle cryo-EM is capable of achieving atomic resolution. Subunits of the homo-hexameric enzyme are colored in magenta, pink, cyan, and three different green hues. [EMD-8194]. (B) Visualization of the density for the cyclic peptide jasplakinolide (yellow) in the cryo-EM map of the non-canonical actin *Pf*Act1 from the malaria-causing parasite *Plasmodium falciparium,* demonstrating the potential of cryo-EM in structure-based drug design. The actin filament is shown in light blue with central subunits colored in dark blue, magenta and cyan. [EMD-3805]. (C) The Volta phase plate has revolutionized the EM field by providing unprecedented contrast for biological specimen without the need of defocusing. The introduction of additional phase shift greatly enhances the phase contrast. Scale bar, 10 nm

In 2015, the structure of β-galactosidase with a molecular weight of 465 kDa was resolved at an overall resolution of 2.2 Å [13] (Fig. 2a). In this structure, the binding mode of the known inhibitor PETG could be studied in molecular detail, highlighting the growing role of cryo-EM in drug discovery. The 2-Å barrier was broken 1 year later with a 1.8-Å structure of the 334-kDa protein glutamate dehydrogenase, currently still the record holder [10].

Higher resolution, however, has not been the only improvement. The proteins studied by single-particle cryo-EM have also become increasingly smaller. The lower size MW limit broke the 100-kDa barrier in 2016 with a structure of the 93-kDa-sized cancer-associated isocitrate dehydrogenase in complex with its inhibitor ML309 at 3.8 Å [10]. This remarkable achievement was surpassed in 2017 with the elucidation of the structure of hemoglobin at 3.2 Å with a size of only 64 kDa. These studies were made possible through the use of the Volta phase plate [14]. This device enhances the phase contrast and thereby improves the signal-to-noise ratio in the low-resolution range and makes it possible to solve the structures of relatively small proteins (Fig. 2c) [15].

Beside these latest developments in hardware, also new software releases have made it easier for scientists to obtain high-resolution structures using cryo-EM. Software for high-throughput data collection is under continuous development, clearly steering in the direction of “on-the-fly” processing. With a number of comprehensive and streamlined image processing suites, such as, for example, Relion, EMAN2, cryoSPARC, or SPHIRE, even scientists with little prior knowledge in image processing can solve cryo-EM structures [16–19]. The field also profits immensely from already available software hitherto used exclusively in X-ray crystallography, e.g. COOT, Rosetta, or similar programs for *de novo* building of atomic models [20].

Apart from this, the emergence of new and the refinement of already established sample preparation methods has also greatly contributed to the resolution revolution, but its detailed description would exceed the scope of this review [21, 22].

## Merits of single-particle cryo-EM as high-resolution structural tool

Various techniques exist in structural biology and should be regarded as complementary. Each technique offers unique advantages and in combination with other methods they offer an optimal tool kit for determining molecular structures. The advantages of single-particle cryo-EM are as follows. First, only little sample is required; 3–5 μl of protein at a concentration of 0.05–5 μM is sufficient for obtaining a structure at near-atomic resolution. However, mostly more protein at the same concentration is needed to screen for optimal freezing conditions (100–200 μl). Second, proteins can be directly prepared and imaged immediately after purification. Therefore, even low-abundant and unstable complexes isolated from endogenous sources can be analyzed. Third, buffer conditions can be almost freely chosen offering a large range of conditions to study the protein of choice. Fourth, while in X-ray crystallography the likelihood of crystal formation drops with increasing molecular weight of the proteins/multiprotein complexes, there is virtually no upper size limit for protein complexes in EM.

Flexible parts of proteins are still a major challenge in structural biology. In X-ray crystallography the protein of interest often has to be extensively re-engineered by removal of loop regions, termini, or glycosylation sites to obtain well-diffracting crystals. It is therefore difficult to study proteins that only properly fold and function when glycosylated [23]. In single-particle cryo-EM, however, the engineering of proteins is normally not needed and the full-length, post-translationally modified proteins can be directly studied. Very flexible parts of the protein average out during image processing and do therefore not impede the analysis. In general, single-particle cryo-EM can deal with a certain amount of sample heterogeneity. Different conformations can be separated and processed using unbiased computational classification procedures, often referred to as *in silico* purification.

## Bridging the gap between cells and atoms

Cryo-EM covers a large range of applications. As described above, it is mostly used to solve high-resolution structures of protein complexes by single-particle cryo-EM. Alternatively, protein structures, especially of small proteins, can be obtained by electron crystallography or micro electron diffraction (micro-ED) (Fig. 1c, e). Cryo electron tomography (cryo-ET) is applied to image proteins and protein complexes in their cellular environment, cellular compartments, whole cells, and tissues [24, 25] (Fig. 1d, e).

Electron crystallography requires 2-D protein crystals. They are imaged under cryogenic temperatures in the TEM and high-resolution information is derived from electron diffraction patterns [26–28]. This technique enabled Henderson and colleagues in 1990 to generate the first high-resolution structure of bacteriorhodopsin [26]. The structure of AQPO determined by electron crystallography reached a resolution of 1.9 Å and allowed the visualization of water molecules and annular lipids interacting with the protein [28]. In general, this method is ideally suited to study the structure of membrane proteins in a lipidic environment [29]. However, *de novo* 2-D crystallization of membrane proteins proved to be difficult, and most of the structures obtained by electron crystallography have only reached medium resolution.

The recent development of micro-ED uses tiny 3-D crystals instead of 2-D crystals. The crystals are smaller than those used for conventional X-ray crystallography, making the technique especially useful for the structure determination of the plethora of proteins that do not readily form large crystals [30, 31]. The crystals are tilted in the TEM and diffraction images are collected at defined tilt angles to later reconstruct the 3-D volume (Fig. 1c). Micro-ED has the potential of becoming a high-throughput method to determine the structures especially of small and difficult-to-crystallize proteins.

In cryo-ET, the specimen is also tilted in the TEM, but images instead of diffraction patterns are recorded at defined tilt angles to later reconstruct the 3-D volume. The spectrum of samples used is broad, ranging from large complexes, such as the nuclear pore complex or envelope viruses, to prokaryotes and thin sections of eukaryotic cells and tissue. Importantly, the specimen should be as thin as possible to allow the passage of electrons and reduce beam damage (Fig. 1d).

Initially developed as an alternative to cryo-sectioning, cryo-focused ion-beam (FIB) milling has been recently shown to be the best method so far to obtain thin slices of larger samples [32]. In this method, a focused ion-beam cuts out a thin lamella with high precision.

The resolution of tomograms is normally not better than 15–20 Å. However, if a tomogram contains several copies of a protein complex, the corresponding subvolumes can be extracted and averaged. This process, called subtomogram averaging, increases the signal-to-noise ratio and the resolution of the reconstruction. For symmetrical particles even near-atomic resolution can be reached using this technique [33, 34].

The combination of FIB milling and cryo-ET is just one example that highlights the integrative nature of cryo-EM. Another demonstrative example is correlative light and electron microscopy (CLEM), in which cryo-EM is combined with fluorescence microscopy, allowing the identification of complexes by means of fluorescence labeling in combination with EM-based subcellular localization studies [35].

In summary, cryo-EM, with all its sub-disciplines and applications, is a very powerful method and greatly extends the toolbox of structural biologists to understand malfunctions in complex biological systems at molecular level.

## Single-particle cryo-EM of biomedically relevant proteins

Single-particle cryo-EM has been very successful in elucidating the structure of a large variety of disease-related macromolecules and cellular machines. In the following, we highlight a number of medically important targets that were structurally studied by cryo-EM.

**Fig. 3:**
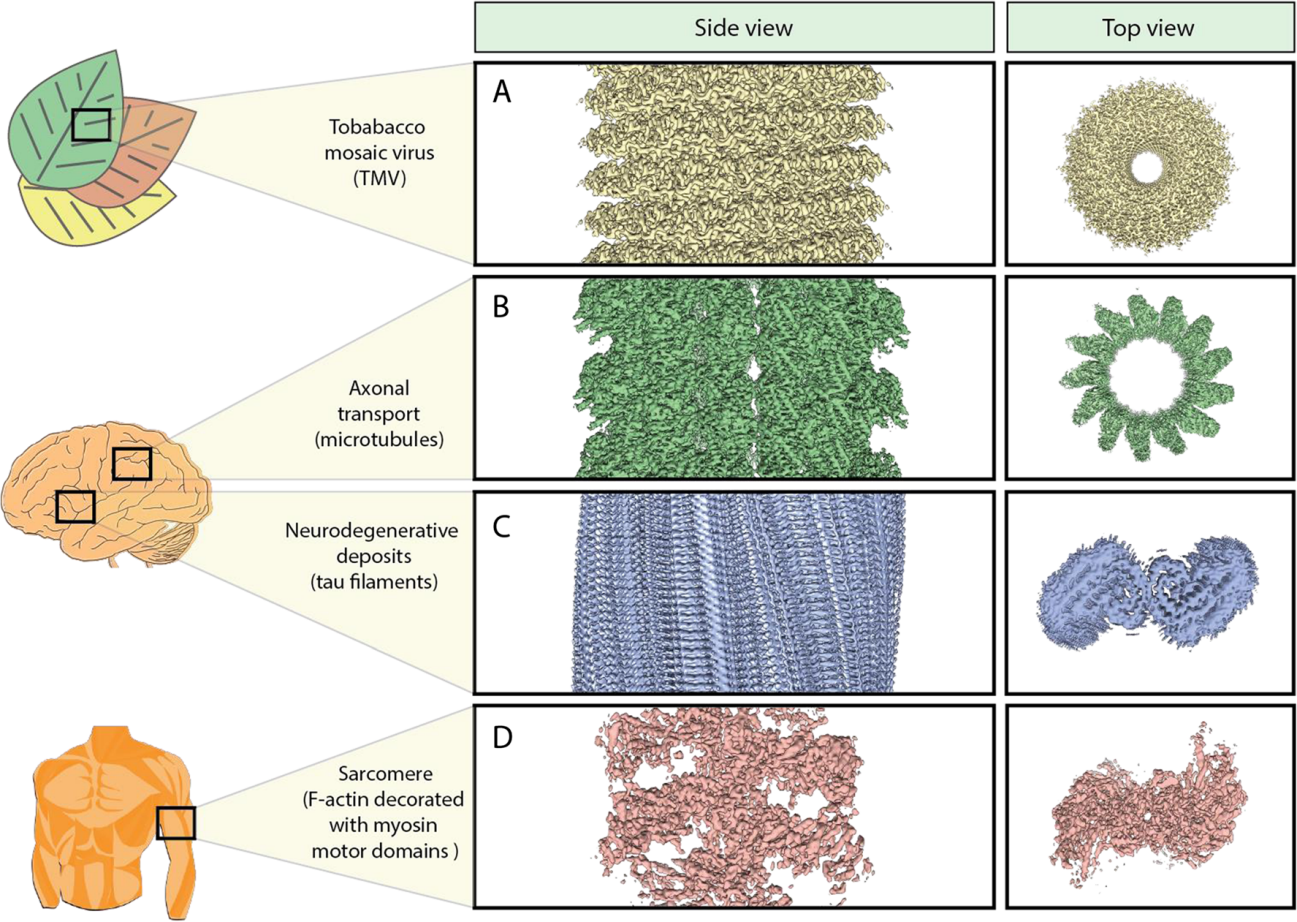
Cryo-EM structures of filamentous proteins and their biomedical relevance. (A) Tobacco mosaic virus is one of the most widespread viruses around the world, being a prime example for plant pathogens that can have far-reaching consequences for the economy as well as food supply. [EMD-2842]. (B) Microtubules are not only core components of the cytoskeleton, but are also essential in axonal transport along neurons, constituting the track for cargo-transporting motor proteins. [EMD-8322]. (C) Tau filaments are neurodegenerative deposits that are found in the brains of AD patients. [EMD-3741]. (D) The interaction of F-actin and myosin filaments is responsible for muscle contraction. Malfunctions can cause myopathies. [EMD-8165]

**Fig. 4:**
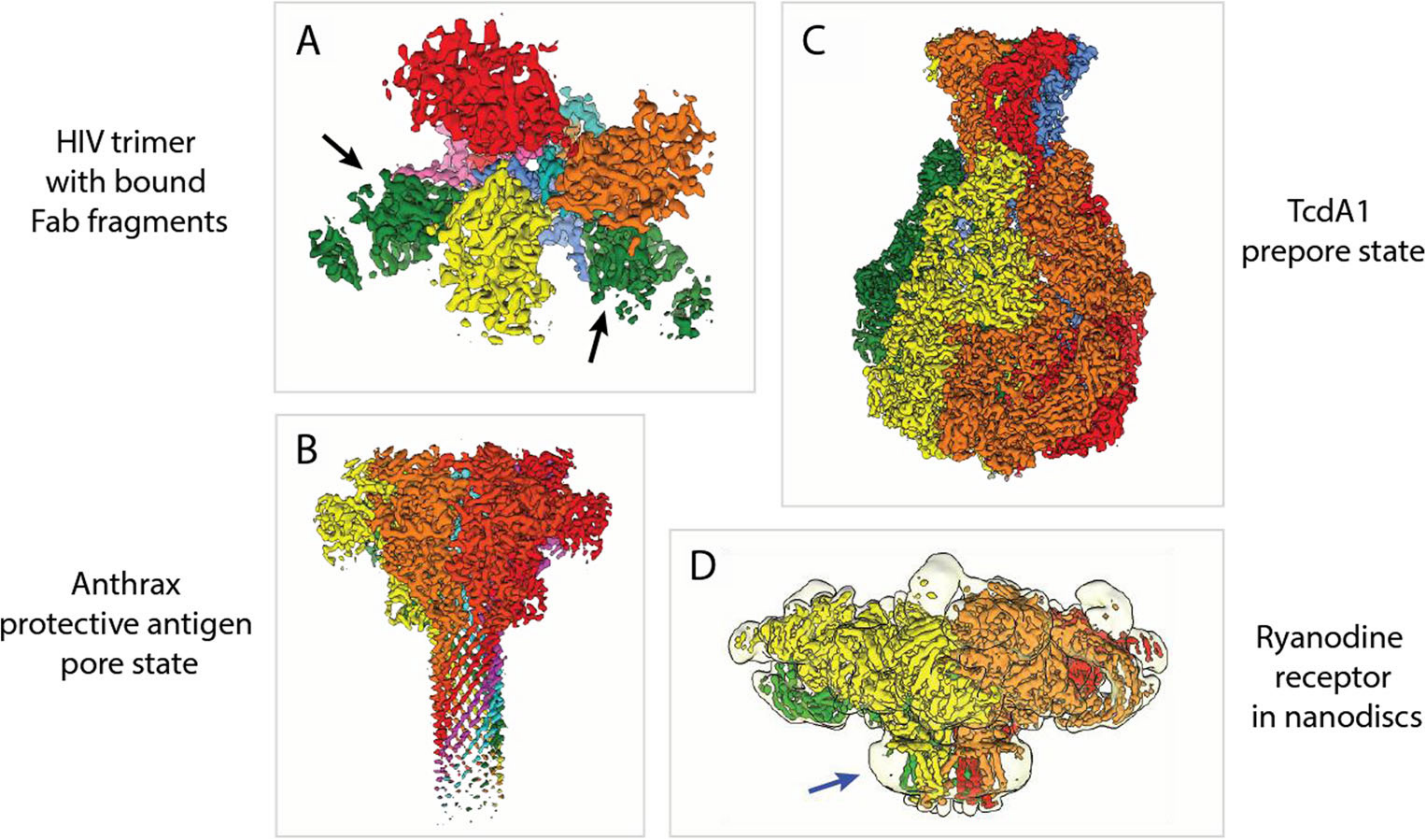
Selected examples of biomedically relevant cryo-EM structures. (A) Cryo-EM structure of the trimeric envelope glycoprotein of HIV. It was solved in complex with two neutralizing antibody Fab fragments. [EMD-3308]. (B) Cryo-EM structure of the anthrax protective antigen pore from *Bacillus anthracis.* [EMD-6224]. (C) Cryo-EM structure of TcdA1 from *Photorhabdus luminescens* in its prepore state. [EMD-3645]. (D) Cryo-EM structure of the ryanodine receptor RyR1. Map at low threshold (transparent) is shown to visualize the nanodisc (blue arrow), which stabilizes the transmembrane helices of RyR1. [EMD-2751]. Individual subunits are depicted in various colors. The hetero-trimeric HIV envelope glycoprotein in A can be divided into the gp120 trimer (yellow, orange, and red) and gp41 trimer (pink, blue, and cyan). Bound Fab fragments (green) are indicated by black arrows. Heptameric anthrax protective antigen pore (yellow, orange, light red, dark red, magenta, cyan, and green) in (B), pentameric TcdA1 (yellow, orange, red, green and blue) in (C), and tetrameric RyR1 (yellow, orange, red, and green) in D

Traditionally, high-resolution cryo-EM structures could only be obtained from large objects with a high degree of symmetry, such as icosahedral viruses [36, 37] or filamentous viruses [38]. While this has been a tedious and difficult process, the structure determination of icosahedral and filamentous viruses by cryo-EM became straightforward after the introduction of the DEDs. Recent important structures include that of the thermally stable Zika virus [39], which belongs to the family of *Flaviviridae* and is linked to congenital microcephaly and the Guillain-Barré syndrome [40]. The 3.7-Å structure provides an important topology map of the virus helping to understand its mechanism of infection and to develop potent vaccines against it. The determination of high-resolution structures of filamentous viruses such as the tobacco mosaic virus (TMV), a plant pathogen, also profited from the new technology (Fig. 3a) [41].

Especially glycoproteins on the surface of envelope viruses are difficult to study in their native state but are of high medical interest as they are the most promising targets for vaccine design. Since they are asymmetrical, envelope viruses are more difficult to study by single-particle cryo-EM. Therefore, these viruses have been mostly analyzed using cryo-ET, giving us a conceptual idea of how viral particles enter cells [42, 43]. With the recent introduction of DEDs, subtomogram averaging became very powerful and enabled the determination of high-resolution structures of the essential trimeric envelope glycoproteins that mediate the cell entry of HIV and Ebola virus. In addition, the structures of these proteins in complex with neutralizing antibodies/corresponding receptors were determined (Fig. 4a) [44–46].

Filamentous actin (F-actin) and microtubules (MTs) are major components of the cytoskeleton and play essential cellular roles in cell division and muscular contraction. They interact with a plethora of proteins and are of central importance for the homeostasis of the cell. Consequently, MT- or F-actin-related disturbances are connected to many diseases, in particular cancer, neurodegeneration, and myopathies [47–49]. Since MTs and F-actin complexes, as well as other filamentous protein complexes, are notoriously difficult to analyze in their respective filamentous state using crystallography, high-resolution structures and thus their understanding at the atomic level has remained enigmatic until recently. Profiting from the new detector technology, near-atomic resolution structures of F-actin and MTs have now been solved (Fig. 2b, 3b) [50, 51]. In addition, recent cryo-EM structures vastly contributed to the understanding of MT dynamics including regulation by MT-associated proteins (MAPs) and revealed the interaction with small molecules that have the potential of becoming anti-cancer drugs [52, 53]. Our lab gained valuable structural insights into the interaction between F-actin and myosin (Fig. 3d) [54]. Furthermore, we solved the structure of the non-canonical actin filaments of *Plasmodium falciparum*, the parasitic microorganism causing malaria, in complex with the naturally occurring cyclic peptide jasplakinolide, exemplifying the potential of cryo-EM for structure-based drug design (Fig. 2b) [55]. We identified that subtle but significant differences are responsible for the inherent instability of the filaments, which is an essential feature for proper host cell invasion of the pathogen.

Alzheimer’s disease (AD), the most common neurodegenerative disease, is characterized by large filamentous deposits in the brain [56]. Single-particle cryo-EM studies allowed for the first time the ability to resolve the structure of aberrantly folded tau filaments isolated from AD patient-derived material to a resolution of 3.4 Å [57]. The structure revealed how two identical protofilaments adopt a cross-β/β-helix structure (Fig. 3c). The filamentous deposits constituting the main component of senile plaques in AD, namely amyloid-β (1–42) fibrils, were also solved by cryo-EM [58]. Based on these findings, novel therapeutics might be tailored to reverse the formation of AD deposits. Relatedly, the highly glycosylated membrane-embedded 130-kDa protease γ-secretase that is responsible for the formation of β-amyloid plaques, has also been studied by cryo-EM [59]. Two hotspots for disease-evoking mutations in presenilin 1, the catalytic component of γ-secretase, have been identified, bringing researchers a step closer to understanding the dysregulation of this crucial protein complex.

Membrane proteins represent the target of ~ 50% of market-approved small-molecule drugs. The determination of their structure is key for directed drug design, but their crystallization is often challenging, hampering a fast progress in drug discovery. In the past 5 years, many high-resolution structures of membrane proteins have been determined by single-particle cryo-EM. Especially for large membrane protein complexes, the technique proved to be superior to X-ray crystallography. Indeed, one of the first near-atomic resolution structures that were solved making use of the new DED technology was that of the transmembrane protein TRPV1, a member of the transient-receptor-potential (TRP) family, which mediates a wide range of sensational input such as pain, taste, pressure, and warmth [60]. The Ca^2+^ channel was also resolved in complex with the spider peptide toxin DkTx and a small vanilloid agonist [61]. The small molecules were clearly resolved in the structures, demonstrating that cryo-EM is indeed able to visualize ligands [62].

The list of all relevant membrane proteins that have recently been solved by cryo-EM is long, and even a very extended review would not suffice to name and describe them in detail. Many structures were determined for a large number of medically important channels and transporters that are closely linked to various diseases [63–65]. In addition, extraordinarily large membrane protein complexes, in particular those ones involved in respiration and photosynthesis, were structurally characterized by cryo-EM [66, 67]. A good example of both a channel and a large membrane protein complex is the ryanodine receptor, a key mediator of calcium release from the sarcoplasmic reticulum, initiating muscle contraction. Three groups in parallel determined the cryo-EM structure of RyR1 giving novel insights into the 2.2-MDa Ca^2+^ channel [68–70]. We reconstituted the channel into small disc-shaped membrane patches, known as lipid nanodiscs, to provide a close-to-native lipid environment (Fig. 4d) [69]. A similar study with TRPV1 reconstituted into lipid nanodiscs revealed a density corresponding to annular lipids interacting with the channel [71]. In recent years, several other lipid mimetic systems based on amphipathic polymers were rediscovered or newly developed, such as amphipols, SMALPs, or the natural scaffold-based saposins, all with the objective to stabilize the respective membrane protein, especially the transmembrane region [72–74].

In regard to pharmacological application of cryo-EM, it should be mentioned that it was recently possible to solve the cryo-EM structure of a B-class G-protein-coupled receptor (GPCR), belonging to the family of the most abundant cell surface receptors and implicated in chronic diseases such as diabetes and obesity. The development and application of the Volta phase plate (see above) [75] was key to solve the structure of these relatively small receptors that were recalcitrant to crystallization so far.

Most multiprotein complexes are highly dynamic in order to execute their respective functions. We have already mentioned above that cryo-EM is able detect and separate different conformational states during image processing of a single dataset. A good example for this is the ATP-synthase. Different conformational states were identified in one dataset and separately processed. The resulting structures represented different conformational states and allowed the correlation of mechanistic information with structural snapshots [76].

A long list of mostly asymmetric macromolecular machines were shown to yield structures, the majority of them reaching near-atomic resolution, including the ribosome [77], spliceosome [78], proteasome [79], dynein/dynactin [80, 81], transcription (pre-) initiation complex [82], inflammasome [83], signalosome [84], and exosome [85], all of them indispensable for cellular performance.

Bacterial toxins, which are self-containing agents, can have profound effects on human health. They are found in a variety of human pathogenic microorganisms, such as, for example, the bacterium *Bacillus anthracis*. The structure of the anthrax protective antigen pore was resolved by single-particle cryo-EM to a resolution of 2.9 Å (Fig. 4b) [86]. The cryo-EM structures of a number of pore-forming toxins that assemble into large pores to disturb essential cellular gradients, usually featuring > 10 subunits, were also determined, giving important insights into conformational changes during membrane insertion [87, 88]. Cryo-EM studies on Tc toxin complexes from *Photorhabdus luminescens* revealed a unique syringe-like injection and translocation mechanism for membrane permeation and delivery of the toxic component into host cells (Fig. 4c) [89, 90]. The energy required for this process is provided by the compaction of an internal entropic spring [91]. Furthermore, cryo-EM and cryo-ET led to unprecedented insights into the architecture of large bacterial secretion systems and the delivery mechanism of important effectors, causing human diseases [92–94].

The large number of various medically relevant protein structures that have been solved by cryo-EM highlights the importance of this technique as a structural tool for biomedical research. Cryo-EM has the potential of becoming the major structural biology technique to study larger protein complexes.

## Challenges and opportunities

The recent revolution in cryo-EM has led to an increased demand for equipment, with smaller labs often having difficulties to afford costly state-of-the-art instrumentation. To counteract this trend, in the last 2–3 years, cryo-EM facilities at many universities and research institutions have been founded. In addition, central facilities have been established to also allow access and professional support for structural biologists without high-end EM equipment. The required time from data collection to the final structure has been tremendously reduced over the past years, facilitated by abovementioned improvements on both hardware and software. Although the resulting throughput is not yet comparable with that in X-ray crystallography, a cryo-EM structure can be obtained in less than a week.

One direction of research that the field is slowly but steadily taking is time-resolved cryo-EM. The aim is to capture short-lived states within non-equilibrium systems to monitor conformational changes over time [95]. These time-resolved “snapshots” could be ultimately used to reconstitute molecular movies of biological processes.

The bottleneck in obtaining atomic structures is now shifting from former technical limitations towards sample production and preparation, requiring strong biochemistry. Recent breakthroughs, in particular the ability to solve the structures of many membrane proteins, have opened up the way for structure-based drug design. Consequently, pharmaceutical companies are becoming increasingly interested in cryo-EM and hopefully invest in its further development. All in all, the field of cryo-EM is constantly evolving and has a bright future ahead.
